# 

**Published:** 1895-07

**Authors:** 


### CONTENTS.

ORIGINAL ARTICLES:
New York College of Veterinary Surgeons . . . . . . . . . . 403
Epizoötic Sore Mouth of Sheep in Montana. By R. N. MEAD and FREDERIC PRIEST . . . . . . . . . . . . . . . . . 412
On the Morbid Histology and Bacteriology of Equine Pneumonia. By CECIL FRENCH, D.V.S. . . . . . . . . . . . . . . 421
Meat and Milk Inspection in Germany. By OTTO NOACK, V.S. . . . . . 423
Investigations Concerning Texas or Southern Cattle-Fever. By N. C. POWELL and F. A. HAMILTON . . . . . . . . . . . . . 427
Belladonna. By S. P. BISHOP . . . . . . . . . . . . . 431
SELECTIONS:
Tuberculous Infection Through the Alimentary Canal . . . . . . . 435
Proprietary Remedies in Veterinary Practice . . . . . . . . . 441
REPORTS OF CASES:
Temporary Paralysis of Peristalsis Following Injuries by Trolley. By W. HORACE HOSKINS . . . . . . . . . . . . . . . . 443
An Obscure Case. By A. W. CLEMENT, V.S. . . . . . . . . . 444
CLINICAL GLEANINGS: . . . . . . . . . . . . . . 446
COMMENCEMENT EXERCISES:
Ohio State School of Veterinary Medicine . . . . . . . . . . 446
University of Pennsylvania—Department of Veterinary Medicine . . . . 446
AMONG THE COLLEGES:
Veterinary Department, Ohio State University—Kansas City Veterinary College—McKillip Veterinary College—Veterinary Department, University of Pennsylvania . . . . . . . . . . . . . . . . . 447
EDITORIALS:
On to Des Moines—Veterinary Titles—A More Confident Public Gradually Developing . . . . . . . . . . . . . . . . . 452
U. S. V. M. A.: . . . . . . . . . . . . . . . . . 455
CORRESPONDENCE — PERSONAL — MARRIAGES AMONG VETERINARIANS — EDUCATIONAL—WORTH YOUR KNOWING—SOCIETY PROCEEDINGS—NEW AND USEFUL VETERINARY INSTRUMENTS—NEW INVENTIONS . . . 457–468

